# Age and Overdose at the Index Attempt Predict Suicide Reattempts After Emergency Admission: A Multicenter Cohort Study

**DOI:** 10.1002/npr2.70127

**Published:** 2026-04-29

**Authors:** Taro Sasaki, Norio Sugawara, Motoharu Furukawa, Hirotaka Iwaki, Shiro Suda, Norio Yasui‐Furukori

**Affiliations:** ^1^ Department of Psychiatry Dokkyo Medical University School of Medicine Mibu Tochigi Japan; ^2^ Department of Psychiatry Jichi Medical University Shimotsuke Tochigi Japan; ^3^ Department of Psychiatry Hachinohe City Hospital Hachinohe Aomori Japan

**Keywords:** age, Cox proportional hazards model, emergency admission, overdose, suicide reattempt

## Abstract

**Aim:**

A history of suicide attempts is a well‐established predictor of future suicidal behavior; however, longitudinal evidence from real‐world emergency settings remains limited. Because age and self‐poisoning/overdose are readily available at emergency presentation and may inform post‐discharge care, we examined whether age and overdose at the index suicide attempt predict medically attended suicide reattempts after emergency admission.

**Methods:**

We conducted a multicenter retrospective cohort study of 1038 patients admitted to emergency departments after suicide attempts between 2020 and 2025 at three tertiary hospitals in Japan (Dokkyo Medical University Hospital, Jichi Medical University Hospital, and Hachinohe City Hospital). Patients were followed longitudinally to identify suicide reattempts requiring medical attention. Time‐to‐event analyses were performed using Kaplan–Meier methods and Cox proportional hazards models. Age (< 40 vs. ≥ 40 years) and sex were included as baseline covariates, and overdose at the index attempt was examined as an explanatory variable. Sensitivity analyses were performed using a stricter definition of overdose (overdose alone).

**Results:**

During follow‐up, 58 medically attended suicide reattempts were identified. Kaplan–Meier analyses showed higher cumulative reattempt risk in females and in patients aged 39 years or younger. In Cox proportional hazards models, age ≥ 40 years was associated with a lower risk of suicide reattempt, whereas overdose at the index attempt was associated with a significantly increased risk of suicide reattempt. These findings were consistent in sensitivity analyses.

**Conclusion:**

Younger age and overdose at the index suicide attempt were independently associated with an increased risk of medically attended suicide reattempt. Assessment of the initial suicide method may help identify patients requiring intensive post‐discharge monitoring.

## Abstract 2

A multicenter cohort of 1038 patients admitted to three Japanese tertiary emergency hospitals after suicide attempts was followed to identify medically attended suicide reattempts. Cox proportional hazards models showed that overdose at the index attempt increased the risk of reattempt, while age ≥ 40 years was protective; findings were consistent in sensitivity analyses.

## Graphical abstract

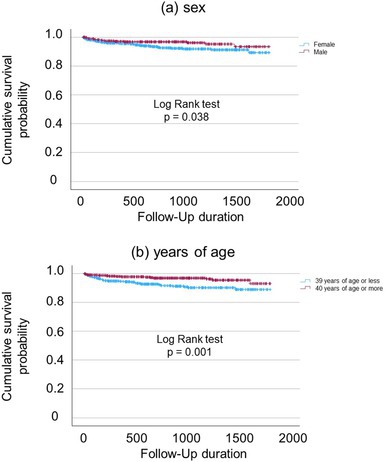

## 1 Introduction

Suicide attempts represent one of the strongest predictors of subsequent suicidal behavior, and individuals with a history of suicide attempts are known to have a markedly elevated risk of future reattempts and suicide death [1, 2]. Large cohort studies and systematic reviews have consistently identified multiple risk factors for suicide reattempts, including persistent suicidal ideation, mood disorders, substance use, and personality pathology [1, 3, 4]. Among these factors, demographic characteristics—particularly age and sex—have been repeatedly associated with patterns of suicidal behavior.

Previous studies have shown that younger individuals are more likely to engage in repeated suicide attempts, whereas older age is more strongly associated with suicide completion [5, 6]. Sex differences have also been widely reported, with females exhibiting higher rates of suicide attempts and reattempts, while males have higher rates of suicide completion [3, 6]. However, accumulating evidence suggests that these sex differences may be partly explained by differences in the choice of suicide methods rather than sex itself [3, 6].

Although multicenter Japanese evidence focusing on suicide reattempts has been reported (e.g., the ACTION‐J study) [7], longitudinal data derived from routine, real‐world emergency settings remain limited. In particular, few multicenter studies have followed patients admitted to emergency departments after suicide attempts to evaluate the risk of subsequent reattempts over time with attention to the prognostic value of the index method. Moreover, the method of suicide attempt—especially overdose—varies substantially by age and sex and may influence subsequent suicidal behavior [8, 9]. Overdose is frequently associated with lower immediate lethality but higher survival, potentially increasing the likelihood of future medically attended reattempts [6, 9]. However, the role of overdose at the index suicide attempt as an independent predictor of reattempt has not been sufficiently examined in multicenter longitudinal analyses.

Therefore, the present study aimed to investigate whether age and overdose at the index suicide attempt are associated with suicide reattempts in a multicenter cohort of patients admitted to emergency departments after suicide attempts. By focusing on simple and readily available clinical variables, this study sought to provide clinically applicable evidence to support risk stratification and post‐discharge suicide prevention strategies in routine emergency care.

## 2 Methods

### 2.1 Study Design and Participants

This study was conducted as part of the DJ project, a structured suicide‐prevention–oriented clinical framework that has been described previously [8]. This multicenter retrospective cohort study included consecutive patients who were transported to emergency departments after self‐inflicted injury or self‐poisoning between March 30, 2020, and March 31, 2025. The study was conducted at three tertiary hospitals in Japan: Dokkyo Medical University Hospital, Jichi Medical University Hospital, and Hachinohe City Hospital. Eligible cases were those clinically classified as a suicide attempt with suicidal intent by the treating physicians (and, where available, emergency psychiatric staff) based on clinical interviews, collateral information, and medical records. Episodes judged to be accidental overdose/poisoning or non‐suicidal self‐injury without intent to die were excluded. Patients were followed longitudinally to identify suicide reattempts during the observation period.

### 2.2 Ethical Considerations

This study was conducted in accordance with the principles of the Declaration of Helsinki. The study protocol was reviewed and approved by the institutional ethics committees of the participating hospitals. Because this was a retrospective observational study using existing clinical data, the requirement for written informed consent was waived, and an opt‐out consent procedure was implemented. All data were anonymized prior to analysis to ensure patient confidentiality.

### 2.3 Clinical Care

All participants received standard‐of‐care emergency management, including assessment of physical condition and psychiatric evaluation and referral as clinically indicated at each participating hospital. The present study was observational and did not evaluate the effects of specific interventions during the index admission or follow‐up.

### 2.4 Outcome

The primary outcome was time to suicide reattempt during follow‐up. Suicide reattempt was defined as a subsequent suicide attempt requiring medical attention after the index emergency admission and clinically judged to involve suicidal intent. Reattempts were identified from medical records at the participating hospitals; events not leading to hospital presentation or presenting to other institutions may not have been captured.

### 2.5 Covariates

Age and sex were included as baseline covariates. Age was dichotomized into < 40 years and ≥ 40 years to provide a pragmatic and clinically interpretable cut‐off and to maintain model parsimony given the limited number of events. The method of the index suicide attempt was classified as overdose (self‐poisoning) or non‐overdose. In the primary analysis, overdose was defined as any suicide attempt in which overdose was included, regardless of whether other methods were used concurrently. Sensitivity analyses were conducted using a stricter definition limited to overdose alone at the index attempt (i.e., excluding concurrent methods such as alcohol use, self‐injury, jumping, or other toxic substances).

### 2.6 Statistical Analysis

Time‐to‐event analyses were performed using Kaplan–Meier survival curves and log‐rank tests. Cox proportional hazards regression models were used to estimate hazard ratios (HRs) and 95% confidence intervals (CIs) for suicide reattempt. Sex, age category, and overdose at the index attempt were entered as explanatory variables. Given the limited number of events (*n* = 58), we used a parsimonious model to reduce overfitting. Statistical significance was set at *p* < 0.05.

## 3 Results

### 3.1 Patient Characteristics and Incidence of Reattempt

Baseline characteristics, distribution of index methods, and characteristics of patients with suicide reattempts are provided in Tables S2 and S3. A total of 1038 patients with suicide attempts were included in the analysis. During the follow‐up period, 58 medically attended suicide reattempts were identified.

### 3.2 Kaplan–Meier Analysis

**FIGURE 1:**
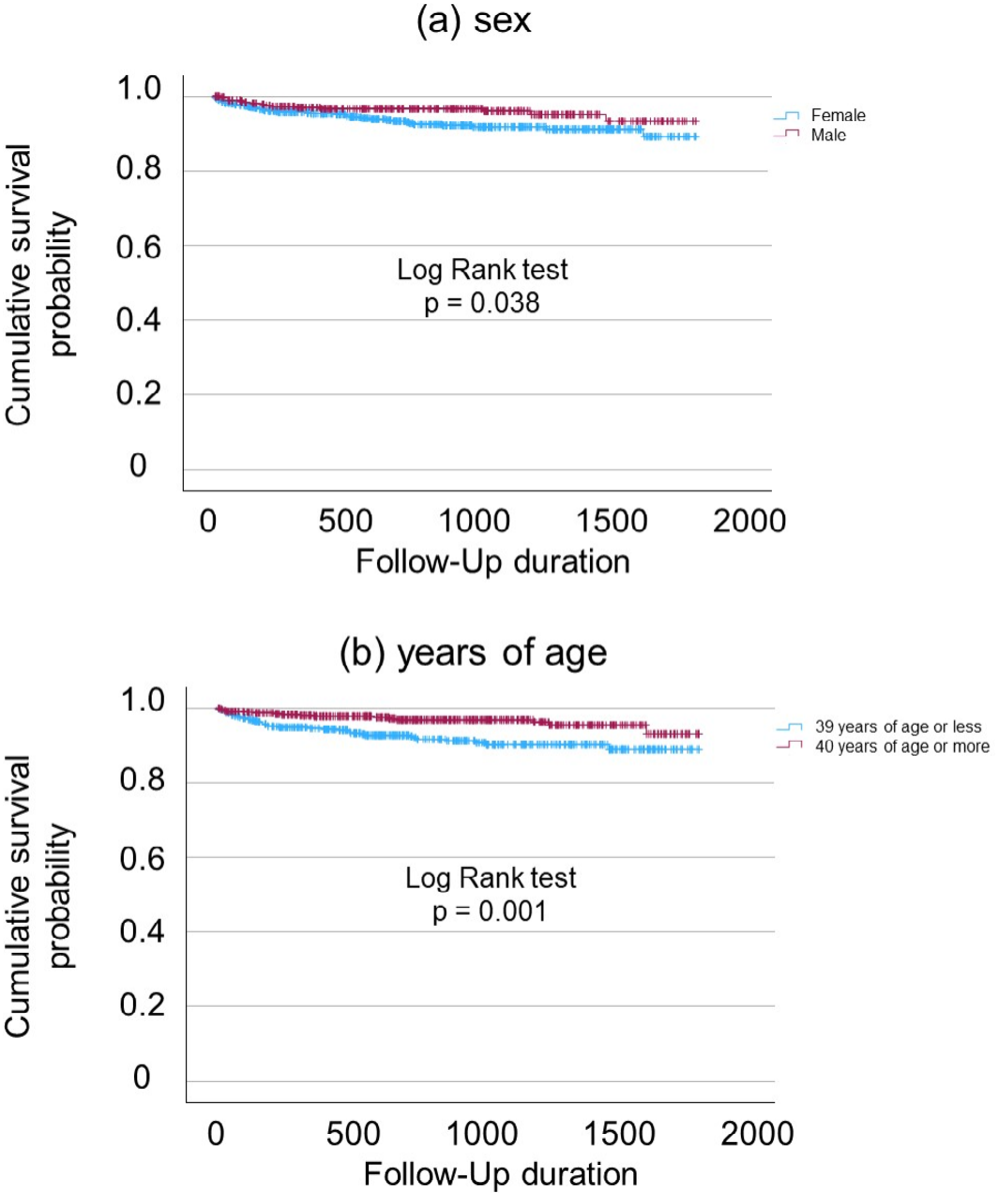
Kaplan–Meier curves for suicide reattempts according to sex and age. Kaplan–Meier survival curves showing the cumulative incidence of suicide reattempts stratified by (a) sex and (b) age group. Female patients showed a significantly higher cumulative risk of suicide reattempts compared with male patients (log‐rank test, *p* = 0.038). Patients aged 39 years or younger had a significantly higher risk of suicide reattempts than those aged 40 years or older (log‐rank test, *p* = 0.001). Follow‐up duration is shown in days.

Kaplan–Meier analyses demonstrated a significantly higher cumulative incidence of suicide reattempts in females compared with males (log‐rank test, *p* = 0.038) (Figure 1a). Patients aged 39 years or younger also showed a significantly higher risk of suicide reattempt than those aged 40 years or older (log‐rank test, *p* = 0.001) (Figure 1b).

### 3.3 Cox Proportional Hazards Models

In Cox proportional hazards models, age ≥ 40 years was associated with a significantly lower risk of suicide reattempt (HR 0.52, 95% CI 0.29–0.94, *p* = 0.030), whereas overdose at the index suicide attempt was associated with a significantly increased risk of suicide reattempt (HR 2.54, 95% CI 1.32–4.90, *p* = 0.005) (Table 1). Sex was not independently associated with suicide reattempt in the multivariable model.

**TABLE 1:** Cox proportional hazards model for suicide reattempts.

Variable	Hazard ratio (HR)	95% confidence interval	*p*
Male sex (vs. female)	0.75	0.41–1.37	0.345
Age ≥ 40 years (vs. < 40 years)	0.52	0.29–0.94	0.030
Overdose at index attempt^a^	2.54	1.32–4.90	0.005

*Note:* Cox proportional hazards regression model including sex, age category, and overdose at the index suicide attempt.

^a^ Overdose was defined as any suicide attempt in which overdose (self‐poisoning with suicidal intent) was included, regardless of whether other methods (e.g., alcohol use, self‐injury, jumping, or other toxic substances) were concurrently used.

In sensitivity analyses restricted to patients whose index suicide attempt involved overdose alone, overdose remained significantly associated with an increased risk of suicide reattempt (HR 2.21, 95% CI 1.22–4.01, *p* = 0.009), and age ≥ 40 years remained protective (HR 0.51, 95% CI 0.29–0.92, *p* = 0.024) (Table S1).

## 4 Discussion

In this multicenter longitudinal cohort study of patients admitted to emergency departments after suicide attempts, younger age and overdose at the index attempt were independently associated with an increased risk of medically attended suicide reattempt. These findings are broadly consistent with longitudinal and register‐based evidence indicating that younger individuals are more likely to repeat suicide attempts, whereas older age is more strongly associated with suicide death [1, 4, 5, 6]. Our results extend this evidence by demonstrating the prognostic value of age and the index method in a real‐world emergency setting across multiple institutions in Japan.

Although Kaplan–Meier analyses showed higher cumulative reattempt rates among females in unadjusted analyses, sex was not an independent predictor in Cox proportional hazards models after adjustment for age and method. This pattern aligns with prior reports suggesting that observed sex differences in suicidal outcomes may be partly mediated by differences in method selection and related clinical features rather than sex itself [3, 6]. Importantly, in the present study, “overdose” refers to self‐poisoning episodes clinically classified as suicide attempts with suicidal intent; accidental overdoses were excluded.

Overdose at the index suicide attempt emerged as a robust predictor of subsequent reattempt, with more than a twofold increase in risk, and this association remained significant in sensitivity analyses restricted to overdose alone. While mixed‐method attempts (e.g., overdose combined with other self‐injurious methods) may reflect higher suicidal intent, such episodes can be more medically lethal and may reduce the opportunity for observed reattempts (survival bias) [6, 9]. Consistent with previous reports from Japanese emergency settings, overdose is frequently observed among younger patients and may reflect access to medication, impulsivity, and ambivalence toward death rather than lethal intent alone [7, 8, 9]. Our findings suggest that overdose at the index attempt may serve as a practical marker of vulnerability to repeated medically attended suicidal behavior.

From a clinical perspective, these results have implications for post‐discharge suicide prevention. At the time of emergency admission, age and the index method are readily available and may help clinicians stratify risk and allocate intensive follow‐up resources. Evidence from Japan, including the ACTION‐J study, supports the importance of identifying risk factors among suicide attempters and providing appropriate aftercare [7]. Our results further highlight that patients who attempt suicide by overdose—particularly younger individuals—may warrant enhanced monitoring and support after discharge.

Several limitations should be considered. First, the multivariable model was intentionally parsimonious (58 events) to reduce overfitting; therefore, residual confounding by psychiatric diagnosis, symptom severity, prior attempt history, substance use, and social factors, as well as post‐discharge psychiatric care, is likely [4]. Second, age was dichotomized for interpretability and model parsimony, which may have resulted in information loss. Third, the overdose category was clinically heterogeneous (e.g., type and dose of ingested substances and co‐ingestion of alcohol), and such details were not available for harmonized analysis across sites. Fourth, psychiatric interventions during the index admission and follow‐up (e.g., diagnosis, pharmacotherapy, psychotherapy, and outpatient follow‐up) were not standardized or measured; therefore, we could not evaluate their effects on reattempt risk, limiting causal interpretation. Fifth, identification of suicide attempts and suicidal intent relied on routine clinical judgment and documentation in acute emergency settings, and misclassification cannot be excluded. Finally, reattempts were defined as suicide attempts requiring medical attention and identified from records at participating hospitals; thus, events not presenting for medical care or presenting to other institutions may have been missed.

## 5 Conclusion

In this multicenter cohort study, younger age and overdose at the index suicide attempt were associated with an increased risk of medically attended suicide reattempt. Assessment of suicide method at initial presentation may provide clinically useful information for identifying patients who require enhanced post‐discharge monitoring.

## Author Contributions

N.Y.‐F. conceived and designed the study. T.S., H.I., S.S., and N.Y.‐F. collected the data. N.S., M.F., H.I., and S.S. supervised the project. N.Y.‐F. and N.S. performed the statistical analyses and drafted the manuscript. All authors critically reviewed the manuscript and approved the final version.

## Funding

The authors have nothing to report.

## Conflicts of Interest

The authors declare no conflicts of interest.

## Supporting information


**Table S1:** Sensitivity analysis of the Cox proportional hazards model for suicide reattempts.
**Table S2:** Baseline characteristics of the study cohort and distribution of index methods.
**Table S3:** Characteristics of patients with suicide reattempts and distribution of reattempt methods.

## Data Availability

The data that support the findings of this study are not publicly available because the ethics committees approved the use of clinical records under an opt‐out procedure without individual consent for public data sharing, and because the dataset contains potentially identifiable clinical and temporal information that cannot be fully anonymized. Therefore, the data cannot be deposited in a public repository. De‐identified data may be made available from the corresponding author upon reasonable request, subject to approval by the relevant institutional ethics committees and completion of appropriate data use agreements.
