# Supplementary material for: Age and Overdose at the Index Attempt Predict Suicide Reattempts After Emergency Admission: A Multicenter Cohort Study
Source: Neuropsychopharmacol Rep. 2026 Apr 29;46(2):e70127. doi: 10.1002/npr2.70127 (PMC13126091; doi:10.1002/npr2.70127)
Supplement: Supplementary file 1 — Table S1: Sensitivity analysis of the Cox proportional hazards model for suicide reattempts. Table S2: Baseline characteristics of the study cohort and distribution of index methods. Table S3: Characteristics of patients with suicide reattempts and distribution of reattempt methods. [file NPR2-46-e70127-s001.docx]

Supplementary Material

Supplementary Table S1. Sensitivity analysis of the Cox proportional hazards model for suicide reattempts

| Variable | Hazard ratio (HR) | 95% Confidence interval | p value |
| --- | --- | --- | --- |
| Male sex (vs female) | 0.73 | 0.40–1.33 | 0.306 |
| Age ≥40 years (vs <40 years) | 0.51 | 0.29–0.92 | 0.024 |
| Overdose at index attempt† | 2.21 | 1.22–4.01 | 0.009 |

Footnote: Sensitivity analysis using a stricter definition of overdose.

†Overdose was defined as overdose alone at the index suicide attempt (self-poisoning with suicidal intent), excluding cases involving concurrent methods such as alcohol use, self-injury, jumping, or other toxic substances.

Supplementary Table S2. Baseline characteristics of the study cohort and distribution of index methods

| Characteristic | Total (N=1,038) | Male (n=402) | Female (n=636) |
| --- | --- | --- | --- |
| Age, years (mean ± SD) | 42.4 ± 20.5 | 47.9 ± 19.7 | 39.0 ± 20.3 |
| Age group <40 years, n (%) | 522 (50.3) | 149 (37.1) | 373 (58.6) |
| Age group ≥40 years, n (%) | 516 (49.7) | 253 (62.9) | 263 (41.4) |
| Male sex, n (%) | 402 (38.7) | — | — |
| Index attempt involved overdose (any), n (%) | 590 (56.8) | 170 (42.3) | 420 (66.0) |
| Overdose alone, n (%) | 493 (47.5) | 134 (33.3) | 359 (56.4) |
| Overdose with concurrent method(s), n (%) | 97 (9.3) | 36 (9.0) | 61 (9.6) |
| Index method: self-injury/cutting, n (%) | 112 (10.8) | 53 (13.2) | 59 (9.3) |
| Index method: jumping from height, n (%) | 101 (9.7) | 37 (9.2) | 64 (10.1) |
| Index method: hanging/strangulation, n (%) | 138 (13.3) | 81 (20.1) | 57 (9.0) |
| Index method: poisoning other than medication, n (%) | 99 (9.5) | 50 (12.4) | 49 (7.7) |
| Index method: other, n (%) | 111 (10.7) | 57 (14.2) | 54 (8.5) |
| Psychiatric diagnosis recorded at index admission, n (%) | 965 (93.0) | 359 (89.3) | 606 (95.3) |

Footnote: Values are presented as n (%) unless otherwise indicated. Percentages in the Male and Female columns are calculated within each sex. Overdose refers to self-poisoning episodes clinically classified as suicide attempts with suicidal intent. Index methods were not mutually exclusive; overdose with concurrent method(s) is a subset of overdose (any).

Supplementary Table S3. Characteristics of patients with suicide reattempts and distribution of reattempt methods

| Characteristic | Reattempt (n=58) | No reattempt (n=980) |
| --- | --- | --- |
| Age, years (mean ± SD) | 33.7 ± 16.9 | 42.9 ± 20.6 |
| Age group <40 years, n (%) | 41 (70.7) | 481 (49.1) |
| Female sex, n (%) | 43 (74.1) | 593 (60.5) |
| Index attempt involved overdose (any), n (%) | 47 (81.0) | 543 (55.4) |
| Overdose alone at index, n (%) | 40 (69.0) | 453 (46.2) |
| Psychiatric diagnosis recorded at index admission, n (%) | 56 (96.6) | 909 (92.8) |
| Reattempt method (among reattempts) | — | — |
| Reattempt method: overdose/self-poisoning, n (%) | 50 (86.2) | — |
| Reattempt method: self-injury/cutting, n (%) | 5 (8.6) | — |
| Reattempt method: jumping from height, n (%) | 2 (3.4) | — |
| Reattempt method: hanging/strangulation, n (%) | 3 (5.2) | — |
| Reattempt method: other, n (%) | 7 (12.1) | — |

Footnote: Reattempts were defined as subsequent suicide attempts requiring medical attention and clinically judged to involve suicidal intent. Values are presented as n (%) unless otherwise indicated; percentages are calculated within each group. Reattempt method categories are not mutually exclusive; a single reattempt may involve multiple methods.
